# Decreased urine uric acid excretion is an independent risk factor for chronic kidney disease but not for carotid atherosclerosis in hospital-based patients with type 2 diabetes: a cross-sectional study

**DOI:** 10.1186/s12933-015-0199-y

**Published:** 2015-04-15

**Authors:** Lian-Xi Li, Ai-Ping Wang, Rong Zhang, Ting-Ting Li, Jun-Wei Wang, Yu-Qian Bao, Wei-Ping Jia

**Affiliations:** Department of Endocrinology and Metabolism, Shanghai Jiao Tong University Affiliated Sixth People’s Hospital, Shanghai Clinical Center for Diabetes, Shanghai Diabetes Institute, Shanghai Key Laboratory of Diabetes Mellitus, Shanghai Key Clinical Center for Metabolic Disease, 600 Yishan Road, Shanghai, 200233 China; Department of Endocrinology, The 454th Hospital of Chinese PLA, Nanjing, 210002 China; Department of VIP, Shanghai Jiao Tong University Affiliated Sixth People’s Hospital, 600 Yishan Road, Shanghai, 200233 China

**Keywords:** Urine uric acid excretion, Type 2 diabetes, Chronic kidney disease, Atherosclerosis, Carotid arteries

## Abstract

**Background:**

The associations between urine uric acid excretion (UUAE) and chronic kidney disease (CKD)/atherosclerosis have not been investigated. Our aims were to investigate the relationships between UUAE and CKD and carotid atherosclerotic lesions in hospitalized Chinese patients with type 2 diabetes.

**Methods:**

This was a cross-sectional study that was conducted with 2627 Chinese inpatients with type 2 diabetes. UUAE was determined enzymatically using a single 24-h urine collection. The subjects were stratified into quartiles according to their UUAE levels. Carotid atherosclerotic lesions, including carotid intima-media thickness (CIMT), plaque and stenosis, were assessed by Doppler ultrasound. Both CKD and carotid atherosclerotic lesions were compared between the UUAE quartile groups.

**Results:**

After adjustment for confounding factors, there was a significant decrease in the prevalence of CKD in the patients with type 2 diabetes across the UUAE quartiles (16.9%, 8.5%, 5.9%, and 4.9%; p < 0.001). Multiple logistic regression analyses revealed that the UUAE quartiles were significantly and inversely associated with the presence of CKD (p < 0.001). Compared with the diabetics in the highest UUAE quartile, those in the lowest quartile exhibited a nearly 4.2-fold increase in the risk of CKD (95% CI: 2.272-7.568; p < 0.001). The CIMT value (0.91 ± 0.22 mm for the diabetics with CKD and 0.82 ± 0.20 mm for the diabetics without CKD, p = 0.001) and the prevalence of carotid plaques (62.1% for the diabetics with CKD and 41.8% for the diabetics without CKD, p = 0.025) were significantly higher in the diabetics with CKD than in those without CKD. However, there was no obvious difference in carotid atherosclerotic lesions across the UUAE quartiles after controlling for the confounding factors.

**Conclusions:**

Decreased UUAE was closely associated with the presence of CKD but not with carotid atherosclerotic lesions in hospitalized Chinese patients with type 2 diabetes. Our results suggest that UUAE is an independent risk factor for CKD in type 2 diabetes. In selected populations, such as patient with type 2 diabetes, the role of uric acid in atherosclerosis might be the result of other concomitant atherosclerotic risk factors, such as CKD.

## Introduction

In recent decades, accumulated clinical and epidemiological studies have confirmed the positive correlation between serum uric acid (SUA) and chronic kidney disease (CKD). For example, a large follow-up clinical trial that included 177,570 patients reported that the subjects in the highest SUA quartile exhibited a 2.14-fold increase in the risk for CKD compared with those in the lowest SUA quartile over 25 years [1].

In contrast, many studies have also investigated the relationship between SUA and atherosclerosis. However, unlike the association between SUA and CKD, which has been repeatedly demonstrated, the correlation between SUA and atherosclerosis remains controversial. For example, a previous study indicated that SUA is an independent risk factor for carotid atherosclerosis in type 2 diabetes [2]. However, another study by Iribarren et al. reported that the correlation between SUV levels and carotid intimal-medial thickness (CIMT) did not remain significant after controlling for other risk factors for atherosclerosis [3]. Similarly, in our previous study, we also found no significant association between SUA levels and atherosclerotic lesions in type 2 diabetes [4].

Furthermore, although a number of studies have assessed the relationships between SUA levels and CKD/atherosclerosis, the associations of urine uric acid excretion (UUAE) with CKD and atherosclerosis have not been investigated in general or diabetic populations. To date, epidemiological data are absent regarding these relationships in patients with type 2 diabetes.

Therefore, the aim of the present study was to examine the associations of UUAE with CKD and carotid atherosclerosis in hospitalized Chinese patients with type 2 diabetes. To our knowledge, this is the first study to investigate the associations of UUAE with CKD and atherosclerosis in type 2 diabetes.

## Methods

### Subjects and study design

The present study was cross-sectional and was partly based on data from our previous studies [4-8]. In brief, from January 2007 to June 2009, 3598 patients with type 2 diabetes who were consecutively hospitalized in our department were observed. All patients received a diabetic diet and avoided excessive purine intake and alcohol consumption after admission to the hospital. Of the 3598 subjects, the UUAE data from 3007 patients were available. The following patients were excluded from the study: those taking any drug that interfered with uric acid metabolism, such as losartan, allopurinol and furosemide; patients with underlying renal disease inconsistent with the diabetes, such as glomerulonephritis and autoimmune kidney disease; and patients who had not undergone carotid ultrasound examination or had incomplete clinical data. Ultimately, 2627 patients were included in the final analyses.

All subjects were interviewed to obtain their histories of hypertension (HTNs), cardio-cerebrovascular events (CCEs), use of lipid-lowering drugs (LLDs), use of antihypertensive agents (AHAs), alcohol consumption and smoking habits. The HTNs, CCEs and smoking and alcohol drinking histories were defined according to the criteria utilized in our previous research [4-9]. This study was approved by the ethics committee of the Shanghai Jiao Tong University Affiliated Sixth People’s Hospital. All subjects completed a written informed consent form before entering the study.

### Physical examination and laboratory measurements

The physical examinations and laboratory measurements have been described in our previous studies [4-9]. Briefly, height, weight, waist circumference, hip circumference, and blood pressure were examined in all patients. Blood samples were obtained after an overnight fast and 2 h after breakfast measurements of glycosylated hemoglobin A1C (HbA1c), fasting plasma glucose (FPG), 2-h postprandial plasma glucose (2 h PPG), fasting C-peptide (FCP), 2-h postprandial C-peptide (2 h PCP), total triglycerides (TTG), total cholesterol (TC), high-density lipoprotein cholesterol (HDL-C), low-density lipoprotein cholesterol (LDL-C), alanine aminotransferase (ALT), creatinine (Cr), serum uric acid (SUA), and C-reactive protein (CRP). Based on the plasma glucose and serum C-peptide levels, the homeostasis model assessment for insulin resistance (HOMA2-IR) and insulin sensitivity (HOMA2 %S) values were calculated using the HOMA2 calculator version 2.2.3 [10,11].

Currently, the simplest and most common approach that is applied in clinical practice to determine the renal excretion of uric acid is the measurement of urinary uric acid output over a 24-h period [12]. Therefore, a single 24-h urine sample was collected, and the 24-h UUAE was determined by enzymatic methods. The 24-h urinary albumin excretion (UAE) was calculated as the mean of the values obtained from three separate early morning urine samples during the period of hospitalization. The estimated glomerular filtration rate (eGFR) was calculated using the following equation for Chinese individuals: eGFR = 175× (serum creatinine)^-1.234^× (age)^-0.179^ (×0.79 if female) [13]. CKD was defined as an eGFR less than 60 mL/min/1.73 m2 and/or an UAE ≥ 300 mg/24 h [14] .

### Ultrasonography measurements

Carotid atherosclerotic lesions including CIMT, atherosclerotic plaque and stenosis were measured by Doppler ultrasound as described previously in detail [5,7,8]. The definitions of CIMT, carotid atherosclerotic plaque and stenosis have also been described in detail in our previous study [5-8]. The reproducibilities of the measurements of the carotid arteries have also been reported in our previous studies [7,8].

### Statistical analyses

SPSS 15.0 for Windows software was used for the statistical analyses. P values <0.05 were considered to be statistically significant. The data are expressed as either the means ± the S.D. or as percentages or medians (interquartile range 25%-75%). One-way ANOVAs with LSDs were used for continuous variables with normal distributions for comparisons across multiple groups. For continuous variables with skewed distributions, Mann–Whitney U tests and Kruskal-Wallis H tests were used. The χ^2^ test was used to compare the prevalence data. The partial correlations were used to determine the relationships between the UUAE and the variables. The general linear model was used to compare the means of the continuous variables and the associations between the UUAE quartiles and CIMT when controlling for other factors. Binary logistic regression analysis was performed to examine the associations of the UUAE quartiles with the presence of CKD, carotid plaque and stenosis.

## Results

### Characteristics of the subjects according to UUAE quartiles

The clinical characteristics of the subjects grouped according to the UUAE quartiles are presented in Table 1. The patients were stratified into quartiles based on the UUAE levels with the cutoff limits of <2235, 2235–2848, 2849–3606, and >3606 μmol/24 h. After controlling for age and sex, the diabetics in the higher UUAE quartiles were more likely to be male, younger, smokers and drinkers; have shorter durations of diabetes (DD); have higher DBP, BMI, WHR, FPG, 2 h PPG, FCP, 2 h PCP, HOMA2-IR, TTG, ALT, and eGFR; and have lower HOMA2-%S, HDL-C and Cr.

**Table 1:** **Characteristics of the subjects**

**Variables**	**Q1 (n = 655)**	**Q2 (n = 658)**	**Q3 (n = 657)**	**Q4 (n = 657)**	**p value**	*p value
UUAE(μmol/24 h)	<2235	2235-2848	2849-3606	>3606	—	—
Male (n, %)	315 (48.1%)	337 (51.2%)	385 (58.6%)	439 (66.8%)	<0.001	<0.001
Age (years)	63 ± 13	60 ± 12	57 ± 12	54 ± 11	<0.001	<0.001
*DD (months)	108(48–168)	84(12–144)	72(12–120)	60(12–120)	<0.001	0.001
Smoking (n, %)	146(22.29%)	148(22.49%)	218(33.18%)	254(38.66%)	<0.001	0.004
Alcohol (n, %)	68(10.38%)	88(13.37%)	105(15.98%)	156(23.74%)	<0.001	0.008
Hypertension (n, %)	376(57.4%)	333(50.6%)	336(51.10%)	345(52.5%)	0.056	0.003
CCEs (n, %)	117(17.90%)	84(12.80%)	86(13.10%)	64(9.70%)	<0.001	<0.001
LLDs (n, %)	170(25.95%)	199(30.24%)	191(29.07%)	253(38.51%)	<0.001	<0.001
AHAs (n, %)	346(52.82%)	296(44.98%)	312(47.49%)	305(46.42%)	0.031	0.004
SBP (mmHg)	133 ± 18	133 ± 18	131 ± 17	131 ± 16	0.015	0.295
DBP (mmHg)	78 ± 9	80 ± 10	81 ± 10	81 ± 10	<0.001	<0.001
BMI(kg/m2)	23.9 ± 3.6	24.4 ± 3.3	25.0 ± 3.2	26.2 ± 3.3	<0.001	<0.001
WHR	0.90 ± 0.07	0.91 ± 0.06	0.91 ± 0.06	0.93 ± 0.06	<0.001	<0.001
*FPG(mmol/l)	7.3(5.9-9.4)	7.4(6–9.6)	7.7(6.2-9.5)	8.3(6.8-10.1)	<0.001	0.009
*2 h PPG(mmol/l)	12.9(9.3-16.4)	13.4(10–16.8)	13.2(10.3-16.5)	14.1(10.8-17.5)	<0.001	<0.001
HbA1C (%)	9.2 ± 2.6	9.1 ± 2.5	9.0 ± 2.4	9.1 ± 2.2	0.836	0.266
*FCP (ng/mL)	1.41(0.82-2.38)	1.59(0.94-2.28)	1.68(1.15-2.41)	1.92(1.31-2.8)	<0.001	<0.001
*2 h PCP (ng/mL)	3.07(1.64-5.14)	3.68(1.98-5.43)	3.86(2.3-5.34)	4.21(2.61-5.63)	<0.001	<0.001
HOMA2-IR	1.4(0.90-2.1)	1.5(1.00-2.1)	1.5(1.10-2.1)	1.8(1.2-2.5)	<0.001	<0.001
HOMA2-%S	71(46.63-111.55)	67.25(48.23-96.23)	65.2(47.45-91.5)	56.1(40.4-81.5)	<0.001	<0.001
*TTG(mmol/l)	1.33(0.89-1.87)	1.40(0.96-2.04)	1.43(1.01-2.08)	1.72(1.17-2.59)	<0.001	<0.001
TC(mmol/l)	4.66 ± 1.13	4.75 ± 1.24	4.66 ± 1.07	4.80 ± 1.14	0.068	0.065
HDL-C(mmol/l)	1.16 ± 0.31	1.15 ± 0.31	1.10 ± 0.29	1.05 ± 0.29	<0.001	<0.001
LDL-C(mmol/l)	3.03 ± 0.95	3.14 ± 1.01	3.08 ± 0.88	3.13 ± 0.95	0.163	0.168
*ALT (U/l)	17(12–25)	18(13–28)	20(14–32)	25(16–41)	<0.001	<0.001
*Cr (μmol/l)	67(55–86)	68(56–80)	66(56–79)	66(56–78)	0.058	<0.001
*SUA (μmol/l)	302(249–370)	314(256–380)	304(255–372)	320(267–380)	0.005	0.087
*UAE (mg/24 h)	11(5.9-45.7)	10.2(6.2-24.8)	9.7(6.3-21.9)	13(7.6-32.8)	<0.001	<0.001
*eGFR (ml/min/1.73 m^2^)	103(80–129)	106(88–129)	111(93–133)	113(98–135)	<0.001	0.046
*CRP (mg/l)	1.16(0.46-3.49)	1.10(0.49-2.77)	1.09(0.48-2.34)	1.15(0.51-2.81)	0.644	0.120

Values are expressed as the mean±S.D, median with interquartile range, or percentages.

*Non-normal distribution of continuous variables.

P-value: The p-values were not adjusted for age and sex for the trend.

*P-value: The *p-values were adjusted for age and sex for the trend.

### Associations between UUAE levels and clinical parameters

The associations between UUAE levels and the clinical parameters are shown in Table 2. After adjusting for age, sex and DD, the partial correlations analyses demonstrated strongly positive correlations of UUAE levels with SBP, DBP, BMI, WHR, FPG, 2 h PPG, FCP, 2 h PCP, HOMA2-IR, TTG, TC, ALT, eGFR, and CRP, and significantly negative correlations between UUAE levels and age (controlling for sex and DD), DD (controlling for age and sex), HOMA2 %S, HDL-C, Cr, and UAE in diabetics.

**Table 2:** **Correlations between UUAE and other parameters in the patients with type 2 diabetes**

**Variable**	**Correlation coefficient**	**P values**
Age	−0.192	<0.001
DD	−0.076	<0.001
SBP	0.044	0.039
DBP	0.121	<0.001
BMI	0.250	<0.001
WHR	0.129	<0.001
FPG	0.079	<0.001
2 h PPG	0.077	<0.001
HbA1C	−0.014	0.517
FCP	0.129	<0.001
2 h PCP	0.127	<0.001
HOMA2-IR	0.111	<0.001
HOMA2 %S	−0.136	<0.001
TTG	0.136	<0.001
TC	0.046	0.031
HDL-C	−0.094	<0.001
LDL-C	0.00	0.991
ALT	0.132	<0.001
SUA	0.033	0.122
Cr	−0.125	<0.001
UAE	−0.087	<0.001
eGFR	0.079	<0.001
CRP	0.064	0.003

All correlation coefficients were calculated after adjusting for age, sex and duration of diabetes.

### Comparison of CKD among the UUAE quartiles

**Figure 1:**
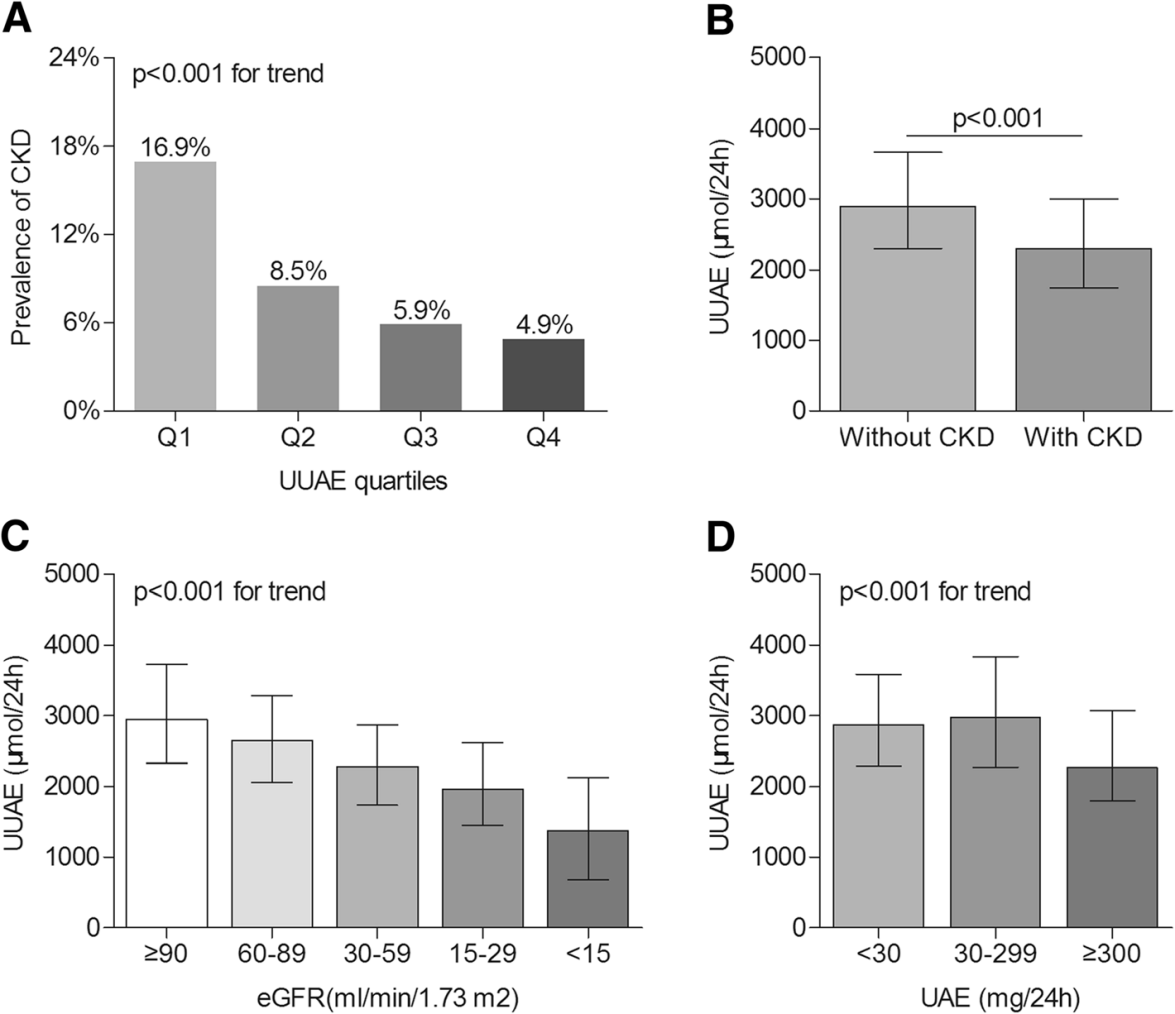
Comparison of CKD among the UUAE quartiles. (**A**) Comparison of the prevalence of CKD among the UUAE quartile groups after adjusting for age, sex, and DD. The P values for the trends were <0.001. (**B**) Comparison of the UUAE levels between the diabetics with and without CKD after adjusting for age, sex, and DD. The P value was <0.001. (**C**) Comparison of UUAE levels among the different eGFR levels after adjusting for age, sex, and DD. The P values for the trends were <0.001. (**D**) Comparison of UUAE levels among the different UAE levels after adjusting for age, sex, and DD. The P values for the trends were <0.001.

Figure 1 demonstrates the comparison of CKD among the UUAE quartiles in patients. After controlling for age, sex, and DD, there was a significantly decreasing trend in the prevalence of CKD in patients across the UUAE quartiles (16.9%, 8.5%, 5.9%, and 4.9%, respectively, p < 0.001 for the trend; Figure 1A). Furthermore, the UUAE levels were obviously reduced in the diabetics with CKD compared to those without CKD (p < 0.001) (Figure 1B). Interestingly, the UUAE levels gradually decreased with the decreases in eGFR (p < 0.001; Figure 1C), and the UUAE levels were significantly decreased in the diabetics with UAEs ≥ 300 mg/24 h compared to those with UAEs <30 mg/24 h and those with UAEs in the range of 30–299 mg/24 h (p < 0.001; Figure 1D).

### Comparison of the carotid atherosclerotic lesions among the UUAE quartiles

**Figure 2:**
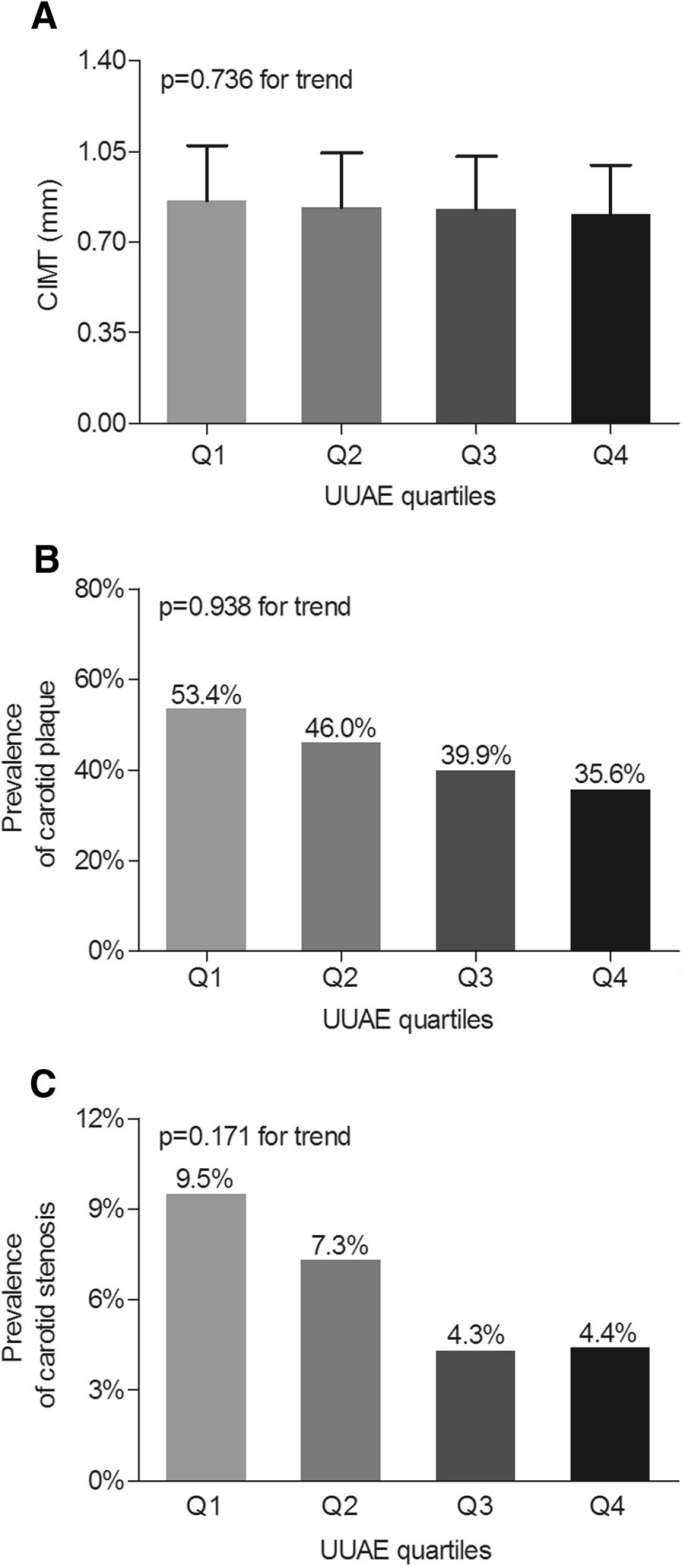
Comparison of carotid atherosclerotic lesions among the UUAE quartiles. (**A**) Comparison of the CIMT values among the UUAE quartile groups after adjusting for age, sex, and DD. (**B**) Comparison of the prevalence of carotid atherosclerotic plaques among the UUAE quartile groups after adjusting for age, sex, and DD. (**C**) Comparison of the prevalence of carotid atherosclerotic stenosis among the UUAE quartile groups after adjusting for age, sex, and DD.

A comparison of the atherosclerotic lesions among the UUAE quartile groups after adjustments for age, sex, and DD is shown in Figure 2. There were no statistical associations between the UUAE quartiles and the carotid IMT values (p = 0.736) and the prevalence of carotid plaques (p = 0.938) and stenosis (p = 0.171) in type 2 diabetes (Figure 2A, B and C).

### Associations of UUAE quartiles with CKD

Table 3 presents the associations between the UUAE quartiles and the presence of CKD in type 2 diabetes. After controlling for age, sex, DD, HTN, smoking, alcohol drinking, and the use of LLDs and AHAs (model 1), the UUAE quartiles were independently associated with a decreased prevalence of CKD (p < 0.001 for trend). After additional adjustments for SBP, DBP, WHR and BMI (model 2) and for ALT, TC, TTG, LDL-C, HDL-C, CRP, HbA1C, FPG, 2 h PPG, FCP, 2 h PCP, HOMA2-IR, and HOMA %S (model 3) and for SUA (model 4), the UUAE quartiles retained an independent association with a decreased prevalence of CKD (all p <0.001 for the trends in model 2, model 3, and model 4).

**Table 3:** **Association of the UUAE quartiles with CKD**

	**ORs (95% CI)**				***P*** **values for trend**
	**Q4**	**Q3**	**Q2**	**Q1**	
Model 1	1	1.225 (0.742-2.021)	1.909 (1.183-3.083)	3.731 (2.375-5.861)	<0.001
Model 2	1	1.437 (0.839-2.460)	2.313 (1.379-3.882)	4.423 (2.695-7.258)	<0.001
Model 3	1	1.860(1.008-3.431)	2.198(1.187-4.070)	4.617 (2.559-8.330)	<0.001
Model 4	1	1.785(0.948-3.359)	1.820(0.967-3.426)	4.147 (2.272-7.568)	<0.001

Model 1: adjusted for age, sex, DD, HTN, smoking, alcohol drinking, and the use of LLDs and AHAs.

Model 2: further adjusted for SBP, DBP, WHR and BMI.

Model 3: further adjusted for ALT, TC, TTG, LDL-C, HDL-C, CRP, HbA1C, FPG, 2 h PPG, FCP, 2 h PCP, HOMA2-IR, and HOMA % S.

Model 4: further adjusted for SUA.

### Comparisons of carotid atherosclerotic lesions between the diabetics with and without CKD

**Figure 3:**
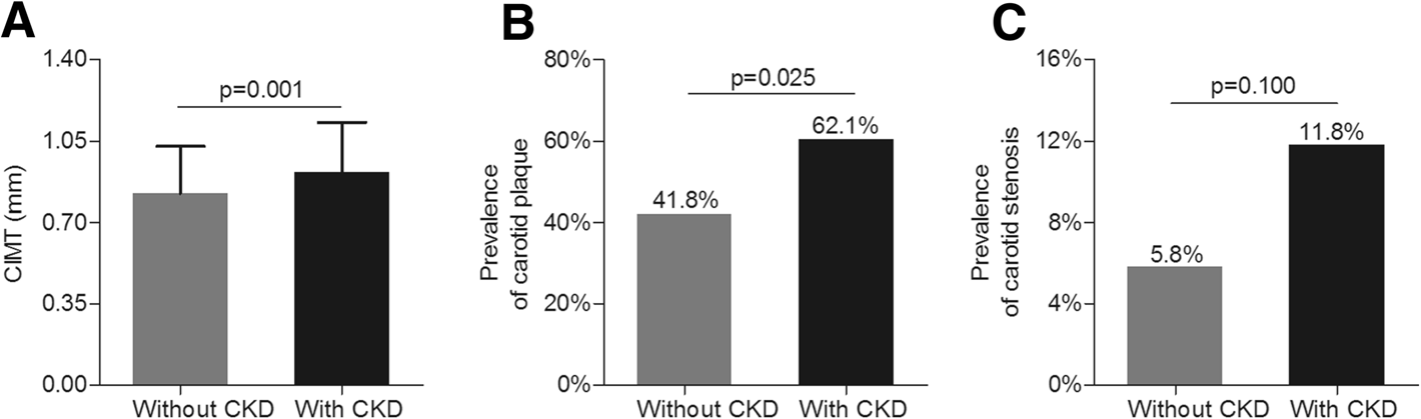
Comparison of carotid atherosclerotic lesions between the diabetics with and without CKD. (**A**) Comparison of the CIMT values between the diabetics with and without CKD after adjusting for age, sex, and DD. (**B**) Comparison of the prevalence of carotid atherosclerotic plaques between the diabetics with and without CKD after adjusting for age, sex, and DD. (**C**) Comparison of the prevalence of carotid atherosclerotic stenosis between the diabetics with and without CKD after adjusting for age, sex, and DD.

The comparisons of carotid atherosclerotic lesions between the diabetics with and without CKD are illustrated in Figure 3. After adjusting for age, sex, and DD, the CIMT values (0.91 ± 0.22 mm for the diabetics with CKD and 0.82 ± 0.20 mm for the diabetics without CKD, p = 0.001) and the prevalence of carotid plaque (62.1% for the diabetics with CKD and 41.8% for the diabetics without CKD, p = 0.025) were significantly higher in the diabetics with CKD than in those without CKD.

## Discussion

Recent evidence has supported uric acid as a potential mediator that is associated with CKD; however, the independence of the association between uric acid and atherosclerosis has been controversial. Furthermore, although a number of studies have focused on the associations of SUA with CKD and atherosclerosis, few investigators have investigated the relationships between UUAE and CKD/atherosclerosis in nondiabetic and diabetic populations.

Therefore, we performed this cross-sectional study of the associations between UUAE and CKD/atherosclerosis in hospitalized type 2 diabetics. Our results strongly suggested that decreased UUAE is an independent risk factor for CKD but not for carotid atherosclerotic lesions in type 2 diabetes.

### The factors controlling UUAE in type 2 diabetes

The mechanisms of controlling UUAE are largely unknown in type 2 diabetes. Generally, UUAE levels are mainly dependent on tubular secretion and postsecretory reabsorption [15]. The renal excretion of uric acid is a complex process regulated by genetic and environmental factors. The primary capacity of the renal excretion of uric acid might be of genetic origin. For example, Emmerson et al. found that both urate clearance and the fractional excretion of uric acid were more similar in monozygotic twins than in dizygotic twins, which indicates that genetic factors exert a key control on the renal excretion of uric acid [16]. Furthermore, a previous study demonstrated that polymorphisms in the N-terminus of the hURAT1 gene are obviously associated with reduced renal uric acid excretion in a German Caucasian population [17]. Therefore, decreased UUAE in type 2 diabetics with CKD might be associated with a genetic predisposition.

In addition to genetic factors, other factors might influence the renal excretion of uric acid. For example, in healthy humans, physiological hyperinsulinemia can reduce urinary uric acid secretion and increase uric acid reabsorption at the tubular levels, which might lead to an increase in SUA levels [15,18]. Therefore, in healthy subjects, UUAE has been shown to be inversely related to insulin resistance and SUA [18]. However, in contrast to healthy subjects, in our present study, the diabetic patients in the higher UUAE quartiles were associated with reduced insulin sensitivity and increased insulin resistance, which theoretically should result in decreased UUAE. Indeed, patients with decreased UUAE are typically associated with impaired renal function and decreased rates of renal filtration, which might be attributed to uric acid-mediated renal damage. Consistent with our hypothesis, we observed an inreased eGFR among the UUAE quartiles. Additionally, because of the inhibitory effects of uric acid reabsorption in the kidney due to high glucose levels, UUAE levels are often increased in patients with diabetes, particularly in diabetes patients with poor glucose control [19,20]. In accordance with this perspective, we observed a positive correlation between UUAE and blood glucose levels, and the blood glucose levels were gradually elevated across the UUAE quartiles in type 2 diabetes.

Therefore, we speculated that decreased UUAE might initially originate from genetic factors in type 2 diabetics with CKD. Reduced UUAE led to impaired renal function, which further aggravated the decreases in UUAE. Thus, diminished UUAE was a risk factor for the development and progression of CKD rather than a consequence of impaired renal function.

### The association between UUAE and CKD

More importantly, we observed a significant inverse association between UUAE and the presence of CKD in type 2 diabetics. A number of investigations have shown that an elevated SUA level is an independent risk factor for CKD in both the general population and the population with type 2 diabetes [1,21-23]. For example, a 5-year follow-up study demonstrated that every 1-SD increment in the SUA level was obviously associated with a 21% increase in the CKD in type 2 diabetic patients [23]. Furthermore, two observational studies that were performed with type 2 diabetes patients revealed that even high-normal SUA was also associated with albuminuria and impaired renal function [24,25]. Consistent with the results of these studies, our results revealed that the prevalence of CKD substantially increased with a decrease in UUAE in type 2 diabetes. Compared with the diabetics in the highest UUAE quartile, the diabetics in the lower UUAE quartiles exhibited a significantly higher risk of CKD. Moreover, the patients with either lower eGFR or higher UAE exhibited lower UUAE levels, which indicated that decreased UUAE might be related to the severity of CKD in type 2 diabetes.

The mechanisms underlying the association between decreased UUAE and CKD remain to be elucidated. However, it has been speculated that the local accumulation of uric acid in the kidney might be one of the mechanisms that causes CKD. Decreased UUAE in type 2 diabetes might indicate that uric acid accumulates in the kidney rather than entering the blood circulation. In an uricase gene-knockout mouse models, Wu et al. found that mouse develop pronounced hyperuricemia, renal tubular crystal deposition and renal failure [26], and these findings indicate the a key role of uric acid in the pathogenesis of CKD. More importantly, uric acid can exert direct effects on renal tubules. A recent experimental study found that uric acid can induce a phenotypic transition termed the epithelial-to-mesenchymal transition (EMT) in renal tubular cells and in the renal tubules of rats with hyperuricemia [27], and this transition plays an important role in pathogenesis of CKD. Furthermore, allopurinol significantly inhibits uric acid-induced phenotypic changes and leads to the amelioration of renal fibrosis [27]. Therefore, uric acid accumulation in the kidney undoubtedly causes the progression of kidney injury and reduced UUAE. Reduced UUAE further aggravates kidney lesions.

### The association of UUAE/CKD with carotid atherosclerotic lesions

In contrast, there are no significant associations between UUAE and carotid atherosclerotic lesions in type 2 diabetes. Although no data about the association between UUAE and atherosclerosis are currently available, debate continues regarding the relationship between SUA levels and atherosclerosis in healthy individuals and patients with diabetes. Several studies have reported that high levels of SUA are associated with carotid atherosclerosis in both healthy and type 2 diabetic subjects [2,28-31]. For example, Fukui et al. found a positive correlation between SUA concentration and CIMT [28]. Similarly, Erdogan et al. also found that increased SUA levels, even within the physiological range, are significantly correlated with increased CIMT independently of other cardiovascular risk factors in healthy adults [29].

However, several cross-sectional and epidemiological follow-up studies, including our own study, have concluded that SUA is not independently associated with atherosclerosis or coronary heart disease [3,4,32-36]. For example, Iribarren et al. reported that the statistically significant association between SUA and CIMT was lost after further adjustments for known risk factors for atherosclerosis [3]. Our own study also found that SUA levels are associated with HTN and metabolic syndrome but not with atherosclerosis in type 2 diabetes [4]. Thus, the observed association between elevated SUA levels and atherosclerosis might be a consequence of concomitant other cardiovascular risk factors, such as HTN and metabolic syndrome [4,37]. Similar to the above studies, we failed to observe an independent association between UUAE and carotid atherosclerosis in type 2 diabetes in this study.

It has been demonstrated that atherosclerotic lesions are accelerated in patients with CKD even in early stages of renal dysfunction primarily due to a wide variety of accompanying traditional and non-traditional cardiovascular risk factors in the CKD population, such as aging, HTN, dyslipidemia, increased oxidative stress, inflammation and vascular calcification [38]. Therefore, it is difficult to determine whether CKD itself is independently related to the increased prevalence of atherosclerosis in patients with CKD. However, recent studies have strongly indicated that CKD itself might be an independent risk factor for atherosclerosis [39-42]. For example, one investigation enrolled 4297 asymptomatic subjects undergoing coronary CT angiography and reported that, after adjustment for confounding factors, early CKD was an independent risk factor for coronary atherosclerosis [42]. Furthermore, a study performed in Chinese patients with type 2 diabetes demonstrated that hypertension, dyslipidemia, and CKD have cumulative effects on the burden of carotid plaques [43]. Given the absence of an association between UUAE and carotid atherosclerosis, we believe that in selected populations, such as those with type 2 diabetes, the pathogenetic roles of uric acid in the development and progression of atherosclerosis might be indirect and mediated by other cardiovascular risk factors, such as CKD, HTN and metabolic syndrome. Therefore, the role of uric acid in the development and progression of atherosclerosis is ambiguous and requires further clarification.

Although multiple risk factor interventions and some medicines, such as liraglutide, can mitigate atherosclerotic lesions in type 2 diabetes, none of the biomarkers that are utilized, such as endothelial function parameters, can predict changes in the progression of atherosclerosis [44,45]. Additional risk factors related to atherosclerosis, such as CKD, should be identified, and early interventions will be useful for reducing future cardiovascular events in type 2 diabetes.

### Limitations

Several limitations of this study must be mentioned. Because the participants were Chinese patients with type 2 diabetes, the results might not be generalizable to other populations. Additionally, due to the cross-sectional nature of this study, we are not able to establish the causal relationship between decreased UUAE and CKD. Prospective studies are needed to determine the relationship between UUAE and CKD. Finally, the examination of UUAE was determined by a single 24-h urine collection rather than three successive collections. Thus, the fluctuations of UUAE could not be minimized. However, when a careful protocol is followed, and the patients strictly follow a standard diet, the difference in 24-h uric acid excretion is very minor [46]. Furthermore, it might be impractical to collect three urinary samples in clinical practice.

## Conclusions

Our results suggest that decreased UUAE is independently associated with the presence of CKD but not with carotid atherosclerosis in type 2 diabetes. Decreased UUAE levels might serve as a simple and useful marker for predicting the occurrence and progression of CKD in type 2 diabetes. In selected populations, such as patients with type 2 diabetes, the role of uric acid in atherosclerosis might result from other concomitant atherosclerotic risk factors, such as CKD. Further study is required to determine whether promoting UUAE represents a new therapeutic target for the prevention of CKD in type 2 diabetes.

## Abbreviations

UUAE: Urine uric acid excretion
CKD: Chronic kidney disease
CIMT: Carotid intima- media-thickness
BMI: Body mass index
WHR: Waist-to-hip ratio
SBP: Systolic blood pressure
DBP: Diastolic blood pressure
DD: Duration of diabetes
CCEs: Cardio- cerebrovascular events
LLDs: Lipid-lowering drugs
AHAs: Antihypertensive agents
SUA: Serum uric acid
UAE: Urinary albumin excretion
eGFR: Estimated glomerular filtration rate
ALT: Alanine aminotransferase
Cr: Creatinine
CRP: C-reactive protein
LDL-C: Low-density lipoprotein cholesterol
FPG: Fasting plasma glucose
2-h PPG: 2-h Postprandial plasma glucose
HbA1c: Glycated hemoglobin A1C
FCP: Fasting C-peptide
2-h PCP: 2-h Postprandial C-peptide
TTG: Total triglycerides
TC: Total cholesterol
HDL-C: High-density lipoprotein cholesterol
